# Chemical Derivatization Strategies for Expanding Small Biomolecule Analysis by MALDI-MS

**DOI:** 10.3390/metabo16090654

**Published:** 2026-09-07

**Authors:** Xintong Hu, Qinxue Wang, Yingying Lin, Jingjing Wan

**Affiliations:** 1 School of Chemistry and Molecular Engineering, East China Normal University, Shanghai 200241, China; 2 Institute of Magnetic Resonance and Molecular Imaging in Medicine, East China Normal University, Shanghai 200241, China

**Keywords:** chemical derivatization, MALDI-MS, biomolecules, spatial metabolomics

## Abstract

Small biomolecules provide important information on cellular metabolism, signaling and disease-associated molecular changes. Matrix-assisted laser desorption/ionization mass spectrometry (MALDI-MS) enables rapid, high-throughput and spatially resolved molecular analysis, but its application to small biomolecules is often limited by poor ionization, low-mass background interference, ion suppression and insufficient structural information. To address these analytical challenges, this review summarizes chemical derivatization strategies that improve MALDI-MS analysis of small biomolecules. The representative strategies are discussed according to reaction mode and target functional group, including solution-phase, on-target, on-tissue, reactive-matrix-assisted and photo-/in-source approaches, with emphasis on amine-, carbonyl-, carboxyl- and double-bond-containing biomolecules. Chemical derivatization improves MALDI-MS performance by selectively modifying target functional groups, introducing charged or ionizable tags, enhancing molecular discrimination and providing additional structural information. These strategies have expanded the detection and annotation of neuroactive amines, carbonyl compounds, glycans, carboxylic acid metabolites and lipid double-bond isomers, while also extending MALDI-MS analysis to other specific functional groups, natural products and drug-related molecules. Overall, chemical derivatization is an important approach for improving the sensitivity, selectivity, structural annotation and analytical coverage of MALDI-MS-based small biomolecule analysis. Future developments should further address reaction selectivity, spatial fidelity, quantitative reliability and confident identification of derivatization products.

## 1. Introduction

Small biomolecules, including amino acids, neurotransmitters, organic acids, and lipids, constitute chemically diverse mediators and products of cellular metabolism and signaling. Alterations in their abundance, chemical state, and spatial distribution may contribute to or reflect pathological processes [1,2]. Accordingly, small-biomolecule analysis has become an important component of life science and disease research, providing valuable information for elucidating disease mechanisms and identifying potential biomarkers [3,4,5,6,7]. However, the detection of small biomolecules remains challenging. Many analytes are present at low concentrations in biosamples and are susceptible to ion suppression and interference from complex biological matrices and background signals. In addition, small biomolecules exhibit broad physicochemical diversity and frequently occur as structural isomers, positional isomers or cis-trans isomers, which increases the difficulty of accurate molecular identification. These issues become even more relevant in in situ tissue analysis, where molecular detection must be achieved without losing spatial information.

Mass spectrometry is a key platform for the analysis of small biomolecules, offering advantages such as high sensitivity, good selectivity, a broad detection range and the ability to provide structural information. Currently, liquid chromatography–mass spectrometry (LC-MS) and gas chromatography-mass spectrometry (GC-MS) are well-established techniques for the qualitative and quantitative analysis of small molecules. However, their performance often depends on analyte-specific extraction, separation, enrichment, or derivatization, particularly for low-abundance, poorly ionizable, nonvolatile, or thermally unstable metabolites [8]. In addition, tissue homogenization and solvent extraction disrupt native molecular distributions, limiting their applicability to spatially resolved analysis [9]. In contrast, matrix-assisted laser desorption/ionization mass spectrometry (MALDI-MS) is characterized by simple sample preparation, rapid analysis, high throughput, and direct analysis of tissue sections and other surface-deposited samples. In MALDI, the matrix absorbs laser energy and assists analyte desorption and ionization, generally enabling soft ionization with limited fragmentation [10].

These analytical characteristics have supported the expansion of MALDI-MS from conventional protein and peptide analysis to a broad range of low-molecular-weight compounds [3,4,5,6,7]. Nevertheless, the direct analysis of small molecules by MALDI-MS remains challenging. Many analytes exhibit poor ionization efficiency because they lack readily ionizable groups. In addition, traditional organic MALDI matrices tend to generate background peaks in the low-mass region, which may interfere with target signals. In complex bio-samples, low-abundance and highly polar compounds are also susceptible to ion suppression. Moreover, structurally similar small molecules, lipid double-bond isomers and glycan isomers are difficult to distinguish using MS1 information alone and generally require tandem MS or complementary evidence for reliable identification. To address these issues, chemical derivatization techniques have been developed. By introducing fixed charges, aromatic structures, laser-absorbing groups, isotope labeling or characteristic fragment tags, these techniques enhance the ionization efficiency of target molecules, alter their mass-to-charge ratio, reduce background interference and improve structural identification capabilities. Chemical derivatization has therefore become an important strategy for expanding the capabilities of MALDI-MS in small biomolecule analysis [11,12,13,14].

At present, several instructive reviews have summarized recent advances in MALDI-MS performance improvement, on-tissue chemical derivatization, reactive matrices and chemical labeling strategies for molecules that are difficult to detect directly by mass spectrometry [3,4,5,6,7]. However, many existing reviews are organized primarily around MALDI-MSI applications, on-tissue derivatization, or specific derivatization formats, whereas a systematic discussion centered on derivatization chemistry for small-biomolecule analysis by MALDI-MS remains limited. In this review, chemical derivatization is considered as a general analytical strategy for expanding small-biomolecule analysis by MALDI-MS, with the discussion organized according to both derivatization mode and target functional group. Particular attention is given to how derivatization chemistry expands the analytical information obtainable by MALDI-MS, including enhanced detection of poorly ionizing molecules, functional-group-selective analysis, structural discrimination of isomers, quantitative analysis, and spatially resolved molecular profiling.

## 2. Analytical Roles of Chemical Derivatization in MALDI-MS

Chemical derivatization is a critical strategy for broadening the analytical scope of MALDI-MS for small molecules. Its significance extends beyond signal enhancement to improving overall analytical performance through targeted modification of analytes. These contributions can be summarized as follows.

Firstly, derivatization can improve detection sensitivity by altering the ionization properties of molecules. Many endogenous small molecules exhibit weak responses in MALDI-MS due to a lack of effective ionizing groups or their high polarity. By introducing a fixed charge, enhancing proton affinity or modulating molecular polarity, derivatization can improve the ionization efficiency of target molecules, thereby extending the detection capabilities of MALDI-MS for low-abundance and weakly responsive small molecules [15,16,17].

Secondly, derivatization can provide additional structural information, and thereby improve the reliability of small biomolecule identification. In many untargeted MALDI-MS workflows, molecular annotation is initially based on accurate m/z values, which alone are often insufficient to distinguish isomers and structurally related compounds. By introducing characteristic mass tags, changing the fragmentation of the target analyte, or generating diagnostic fragments, derivatization can transform structural differences that are difficult to distinguish directly into detectable information, thereby improving the ability to identify molecular structures [13,18,19].

Furthermore, selective derivatization reactions designed for different functional groups enable MALDI-MS to extend from non-selective molecular detection to the analysis of specific functional-group-defined molecular classes. Various chemical structures, such as amino, carbonyl, carboxyl and lipid double bonds, can be selectively labeled through corresponding reactions, opening up new possibilities for the analysis of specific metabolic networks, lipid isomers and small bioactive molecules [16,20,21,22,23].

In recent years, derivatization strategies have further advanced toward in situ spatial analysis and workflow integration. On-tissue derivatization enhances the detection of target molecules while preserving their spatial distributions, whereas reactive matrices and photo- or in-source derivatization integrate chemical reactions more closely with the MALDI-MS workflow, thereby improving analytical efficiency and simplifying the overall procedure.

Overall, MALDI-MS derivatization has evolved from strategies primarily aimed at signal enhancement into versatile chemical tools that support sensitive detection, improved structural discrimination, functional-group-selective analysis, and spatially resolved characterization. These advances have substantially expanded the applicability of MALDI-MS to the systematic analysis of small molecules in complex biological samples.

## 3. Functional-Group-Targeted Derivatization Strategies for the Analysis of Small Biomolecules by MALDI-MS

### 3.1. Implementation Modes of Derivatization-Assisted MALDI-MS Analysis

According to where the reaction occurs and how it is coupled to mass spectrometric detection, MALDI-MS derivatization strategies for small molecules can be broadly categorized into solution-phase/on-target derivatization, in situ derivatization on tissue surfaces, reactive matrix-assisted derivatization, and photo-/in-source derivatization.

Solution-phase derivatization is performed before sample deposition or mass spectrometric detection by allowing the derivatization reagent to react with the target molecule in solution, followed by MALDI-MS analysis of the resulting derivative. This approach has been widely used in conventional small-molecule derivatization studies, with advantages such as easily controllable reaction conditions, high labeling efficiency and suitability for validation with standards [24,25]. However, it generally requires additional sample-handling steps and cannot preserve the native spatial distributions of analytes in tissue sections. In comparison, derivatization performed directly on the MALDI target plate can reduce sample-transfer and purification steps, minimize sample loss, and simplify sample handling [6,7,26].

With the advancement of MALDI-MSI, in situ derivatization on tissue surfaces has become an important strategy for spatially resolved small-molecule analysis. In this approach, derivatization reagents are deposited onto tissue sections, where they react with target molecules before MALDI-MS acquisition, thereby enabling the detection and imaging of the resulting derivatives [3,16,22,27]. Unlike solution-phase or on-target derivatization, on-tissue derivatization is designed to retain the spatial distributions of analytes. Its analytical reliability, however, depends strongly on reaction efficiency, reagent-deposition uniformity, tissue morphology, and analyte diffusion during reagent application and incubation.

To further streamline and integrate the derivatization workflow, researchers have developed reactive matrix-assisted derivatization strategies. This approach combines the functional properties of the MALDI matrix with the ability to react with specific functional groups, enabling a single material system to simultaneously participate in the chemical labeling of analytes, the absorption of laser energy and the ionization process [12,15,26,28,29]. In recent years, photo-induced and in-source derivatization have further promoted the integration of the derivatization process with the MALDI detection workflow. This strategy utilizes light/laser irradiation or reactive species within the ion source to induce rapid reactions, thus reducing the extra incubation steps in conventional derivatization processes while strengthening the control of the reaction temporally and spatially [24,30,31,32,33,34].

Together, the various derivatization approaches represent the evolution of small-molecule analysis by MALDI-MS from offline chemical labeling toward in situ, integrated and rapid reaction systems. Subsequent sections will focus on different target functional groups to discuss how these derivatization strategies improve the sensitivity, selectivity and structural elucidation capability in the detection of specific small molecules.

### 3.2. Derivatization of Amine-Containing Biomolecules

**Figure 1:**
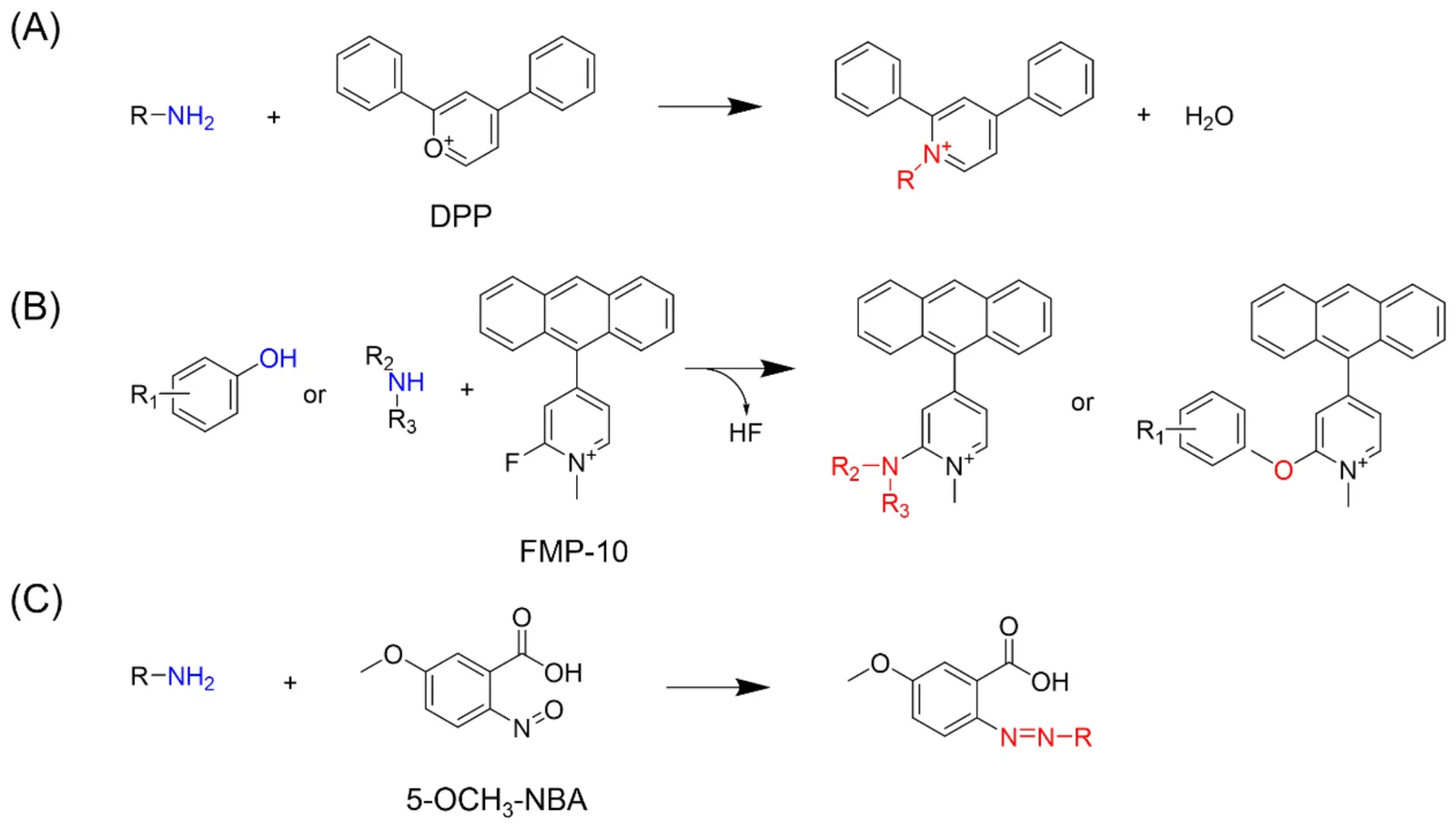
(**A**) Reactive matrix-assisted derivatization of primary amines with 2,4-diphenylpyrylium (DPP). DPP was spray-applied to the sample surface from a methanolic solution containing triethylamine (TEA) at a nozzle temperature of 80 °C, followed by incubation in 50% methanol (MeOH) vapor for 15 min [15]. (**B**) 4-(anthracen-9-yl)-2-fluoro-1-methylpyridin-1-ium iodide (FMP-10)-mediated derivatization of amine- and phenol-containing analytes. FMP-10 (4.4 mM in 70% acetonitrile (ACN)) was spray-applied to the sample surface at a nozzle temperature of 80 °C [12]. (**C**) In-source photoderivatization of primary amines with 5-methoxy-2-nitrobenzaldehyde (5-OCH_3_-NBA). 5-OCH_3_-NBA was prepared in ACN/ethanol/formic acid/water = 84:13:0.3:2.7 (*v*/*v*/*v*/*v*), with titanium dioxide serving as the photocatalytic matrix under nanosecond laser irradiation [32]. The target reactive functional groups in the analytes are highlighted in blue, and the corresponding derivatized moieties in the products are highlighted in red.

Amine-containing biomolecules are widely distributed in biological systems. Many of these molecules are highly polar, present at low abundance, structurally similar, and poorly responsive in direct MALDI-MS analysis, making them susceptible to matrix background interference and ion suppression in complex biological samples. Pyrylium salt-based derivatization strategies have progressively evolved from improving the detection of amine-containing biomolecules to enabling their spatial visualization by MALDI-MSI. Shariatgorji et al. proposed that the 2,4-diphenylpyrylium (DPP) reactive matrix (Figure 1A) selectively derivatizes primary amines to form positively charged pyridinium salt derivatives, thereby enhancing the MS response of primary amine neurotransmitters [15]. This strategy was further extended to brain tissue sections, enabling visualization of neurotransmitter distributions. Subsequently, bromopyrylium (Br-TPP) was developed through bromine substitution on the pyrylium salt scaffold, endowing the derivatives with characteristic bromine isotope peak patterns. These isotopic characteristics further improve the confidence of molecular identification in MALDI-MSI [35]. Unlike DPP and Br-TPP, which mainly target primary amines, 4-(anthracen-9-yl)-2-fluoro-1-methylpyridin-1-ium iodide (FMP-10) can react with a variety of nucleophilic functional groups, including primary amines, secondary amines and phenolic hydroxyl groups, introducing a permanent positive charge into the derivatized products (Figure 1B). These features expand the chemical coverage of neuroactive molecules and facilitate comprehensive spatial mapping of neurotransmitter networks by MALDI-MSI [12].

Collectively, these pyrylium-based strategies highlight that reagent selection should be guided by the intended analytical objective. DPP provides an effective approach for the selective analysis of primary amines, whereas Br-TPP improves molecular identification confidence by introducing diagnostic isotope patterns. In comparison, FMP-10 expands the detectable chemical space through its broader reactivity toward nucleophilic groups; however, this increased coverage may also introduce additional challenges for structural annotation.

Beyond the development of individual derivatization reagents, subsequent studies have focused on improving workflow reproducibility and expanding applications toward more complex biological systems. Standardized on-tissue derivatization protocols have been reported for brain neurochemical analysis, enabling the investigation of diverse molecular classes, including amino neurotransmitters and nucleoside neuromodulators, as well as integration with spatial multimodal analysis [36,37,38,39]. These studies demonstrate that amine-containing derivatization has progressed from enhancing the signals of selected analytes to supporting broader spatial neurochemical profiling and integration with complementary molecular information.

In addition to reactive-matrix approaches, photochemical derivatization has provided another route for the MALDI-MS analysis of amine-containing small biomolecules. Photoderivatization reagents, represented by 2-nitrobenzaldehyde (NBA) and its structural analogues, generate reactive intermediates upon laser irradiation and undergo rapid reactions with primary amines. NBA-based photochemistry was initially applied to primary amine sites in proteins and neuropeptides. Through structural modification of the NBA scaffold by introducing functional substituents, this strategy was further extended to primary amine-containing metabolites, enabling enhanced detection and molecular identification by MALDI-based approaches [24,30,31]. Compared with conventional spray-incubation tissue surface derivatization, NBA-based photoderivatization offers faster reaction kinetics, reduced analyte diffusion, and easier integration into the MALDI-MS workflow. This approach is therefore better suited to the rapid in situ analysis of low-abundance amine-based small molecules.

Building on NBA-based nanosecond photochemical derivatization, Lin et al. further optimized both the structure of the derivatization reagent and the MALDI matrix [32]. Substituent screening identified 5-methoxy-2-nitrobenzaldehyde (5-OCH_3_-NBA) as a more efficient reagent, which was combined with the photocatalytic inorganic matrix TiO_2_ P25 to establish an in-source photoderivatization-enhanced MALDI-MS platform (Figure 1C). Nanosecond laser irradiation induced rapid covalent labeling of primary amine-containing neurometabolites during MALDI analysis, while TiO_2_ P25 reduced low-mass background interference and supported analyte desorption and ionization. This integrated strategy avoided prolonged pre-incubation, reduced sample consumption, and coupled derivatization more closely with MALDI-MS acquisition. The incorporation of a photocatalytically active inorganic nanomatrix further combined laser-triggered chemical labeling with low-background desorption/ionization, advancing NBA-based photochemistry toward nanomatrix-assisted in-source derivatization.

These studies indicate that amine derivatization has become an important route for improving the MALDI-MS analysis of neuroactive small molecules. Together, these strategies illustrate the transition of amine derivatization from improving the detectability of individual neuroactive molecules toward more comprehensive and spatially resolved neurochemical profiling. Pyrylium-based derivatization improves ionization efficiency and molecular specificity through charge tagging and isotope encoding, whereas NBA-based photoderivatization provides unique advantages by integrating derivatization with MALDI laser excitation, thereby reducing the need for prolonged incubation and minimizing analyte diffusion during in situ analysis. Nevertheless, the analysis of amine-containing molecules remains challenging because different amines vary substantially in reactivity, ionization behavior and biological abundance. Further work is therefore needed to improve quantitative calibration and to enable broader profiling of multiple neurochemical classes.

### 3.3. Derivatization of Carbonyl-Containing Biomolecules

Carbonyl compounds, which include aldehydes, ketones, steroids, lipid oxidation products, the reducing ends of glycan chains, and various reactive carbonyl metabolites, are extensively involved in processes such as glucose metabolism, hormonal regulation, oxidative stress, inflammatory responses and metabolic reprogramming in disease. Compared with other functional groups, carbonyl groups exhibit higher chemical reactivity and can be specifically captured via selective condensation reactions.

**Figure 2:**
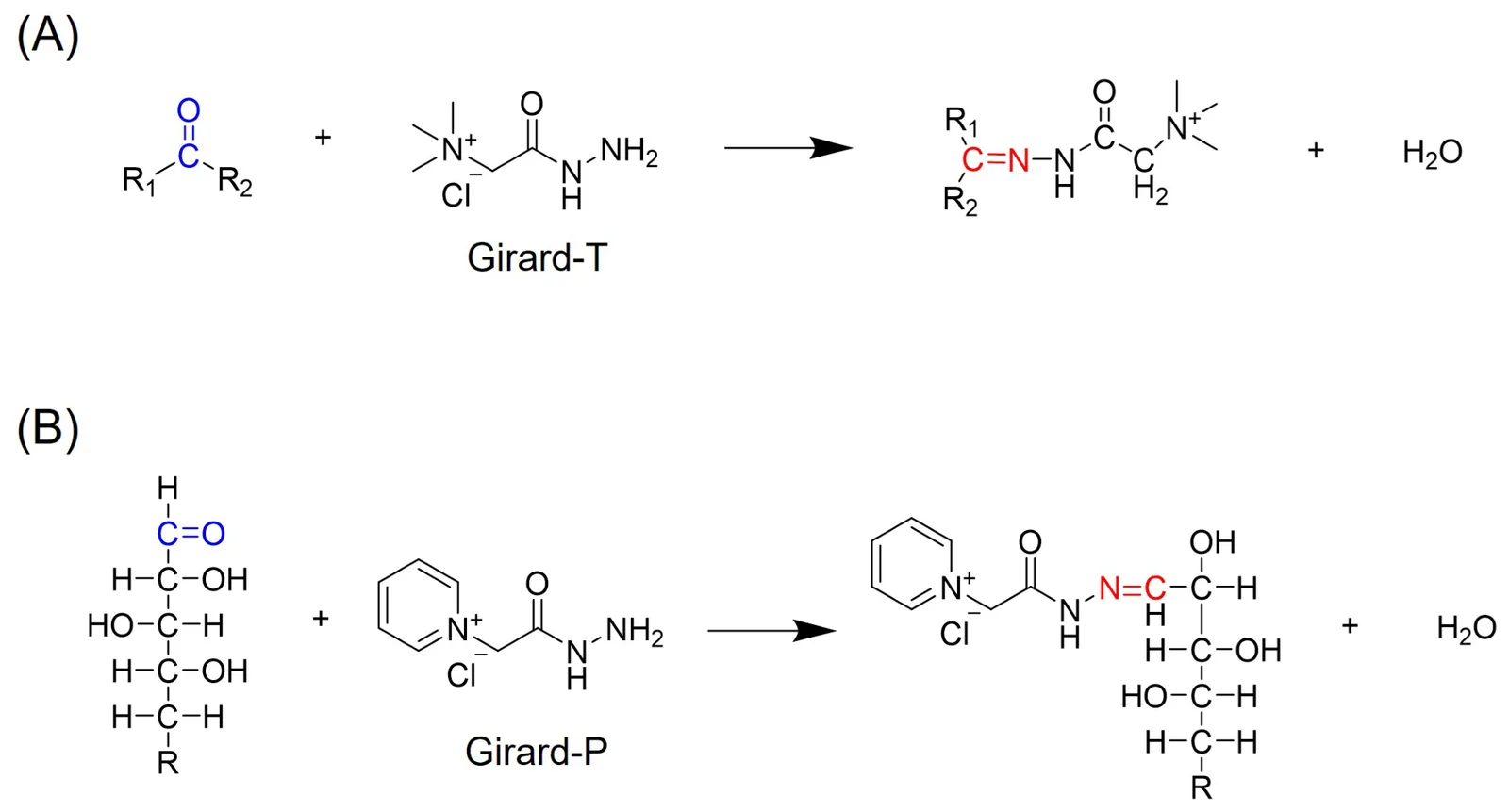
(**A**) Girard’s reagent T (Girard T) derivatization of carbonyl-containing steroids through hydrazone formation. The reaction was performed on the sample surface in MeOH containing 0.2% trifluoroacetic acid (TFA) at 40 °C for 60 min in a humidified environment [40]. (**B**) Girard’s reagent P (Girard P) derivatization of the reducing ends of N-glycans. Girard P (10 mg/mL in 50% MeOH containing 10% acetic acid) was spray-applied to the sample surface at a nozzle temperature of 30 °C and a tray temperature of 45 °C, followed by incubation in 40% acetic acid vapor for 30 min [41]. The target reactive functional groups in the analytes are highlighted in blue, and the corresponding derivatized moieties in the products are highlighted in red.

Early MALDI-MS derivatization of carbonyl compounds focused primarily on hydrazide moieties and Girard-type reagents. The hydrazide moieties in Girard’s reagent P (Girard P) and Girard’s reagent T (Girard T) can react with carbonyl groups such as aldehydes and ketones. The resulting pyridinium or quaternary ammonium salts facilitate ion formation and produce stronger mass spectrometric signals for carbonyl compounds that are otherwise poorly detected by MALDI-MS. Flinders et al. utilized hydrazine-based derivatization reagents for the detection of carbonyl compounds in MALDI-MSI, boosting the mass spectrometric response of aldehyde and ketone molecules [21]. Cobice et al. treated neutral steroids such as corticosterone with Girard T reagent (Figure 2A), enabling MALDI-MSI-based detection and quantitative analysis of weakly ionized steroids in brain tissue subregions [40]. In addition to aldehydes, ketones and steroids, carbonyl derivatization has also been extended to carbohydrate and glycan analysis. Many glycan chains, particularly N-glycans, possess a reducing-end carbonyl group that can react with hydrazide-based reagents. Zhang et al. used Girard’s reagent P for on-tissue derivatization of N-glycans in formalin-fixed paraffin-embedded tissue sections, thereby improving N-glycan ionization and enhancing detectable N-glycan signals in MALDI-MSI (Figure 2B). In this strategy, Girard’s reagent P introduces a positively charged pyridinium moiety into released N-glycans, which improves the positive-ion response and expands detectable glycan coverage [41]. Related MALDI-MSI studies without Girard P derivatization have further shown that spatial N-glycan profiling can reveal tissue-type- and disease-associated glycosylation heterogeneity [42,43,44]. Therefore, carbonyl derivatization has expanded from small carbonyl metabolites and steroids to glycan-related spatial analysis, providing a useful tool for studying disease-associated glycosylation changes.

Kaya et al. used on-tissue chemical derivatization coupled with MALDI-MSI to achieve spatial analysis of carbonyl- and carboxyl-containing metabolites in brain tissue, demonstrating the potential of functional group-selective derivatization for mapping tissue metabolic networks [22]. To further improve workflow integration, reactive matrix-assisted carbonyl derivatization strategies have also been developed. Lodén et al. proposed hydrazide-based reactive matrices for the sensitive detection of aldehydes and ketones. This strategy incorporates carbonyl-reactive functionality into a MALDI matrix, enabling carbonyl labeling and desorption/ionization to proceed within a unified reagent system [28]. It further reflects the development of carbonyl derivatization from conventional external derivatization reagents toward functionalized reactive matrix systems.

Regarding molecular annotation, the parallel derivatization strategy has further enhanced the reliability of carbonyl metabolome analysis. In 2025, a study employed Girard T and Girard P for parallel carbonyl derivatization of tissue sections. By comparing reagent-specific mass shifts and evaluating the consistency of spatial distributions, the study improved the reliability of carbonyl metabolite identification [23]. Compared with single-derivatization methods, this strategy provides additional structural validation information, helping to reduce potential misannotations in complex tissue samples. In addition to direct carbonyl labeling, expanding the range of derivatizable molecules through pre-reaction chemical transformations represents a key development in carbonyl analysis. Wang et al. proposed a strategy involving on-tissue chemical oxidation followed by derivatization, in which hydroxyl metabolites are pre-oxidized to form carbonyl structures, followed by surface derivatization of the tissue using Girard P, thereby enabling the spatial detection of molecules that would otherwise be unable to participate directly in carbonyl reactions [45]. Similar strategies have been further extended to the analysis of complex metabolites such as monosaccharide isomers and oxylipins, improving the ability to distinguish structurally similar molecules and broadening the scope of carbonyl derivatization in the study of metabolic heterogeneity [18,46].

Carbonyl derivatization strategies have progressively addressed key challenges in the MALDI-MS analysis of carbonyl-containing molecules, including inefficient ionization, spatial characterization, and molecular annotation. Girard-type reagents primarily enhance the detection of weakly ionizing carbonyl compounds through charge introduction, whereas on-tissue derivatization approaches extend carbonyl labeling to spatially resolved tissue analysis. Reactive matrix-assisted strategies further integrate derivatization with MALDI acquisition, simplifying the analytical workflow. In addition, parallel Girard-type derivatization using different reagents provides complementary mass-shift and spatial distribution information, thereby improving confidence in carbonyl metabolite identification. Despite these advances, reliable carbonyl metabolome analysis remains challenging because increased detection coverage must be accompanied by confident discrimination of authentic derivatization products from by-products and false-positive assignments. Combining high-resolution MS/MS, ion mobility, validation with standards and database development will be important for more systematic carbonyl submetabolome annotation.

### 3.4. Derivatization of Carboxyl-Containing Biomolecules

Carboxylic acid compounds are widely present in biological systems, including fatty acids and organic acids. They play a significant role in central metabolism and lipid metabolism, and are important targets in metabolomics research. As carboxyl groups are acidic and readily deprotonated, many carboxylic acid molecules exhibit a weak response in the positive ion mode of MALDI-MS, while in the negative ion mode they are prone to interference from salt ions, tissue matrix and background signals in the low-mass region. Direct MALDI-MS analysis is particularly challenging for low-molecular-weight, highly polar or volatile molecules, such as short-chain fatty acids.

**Figure 3:**
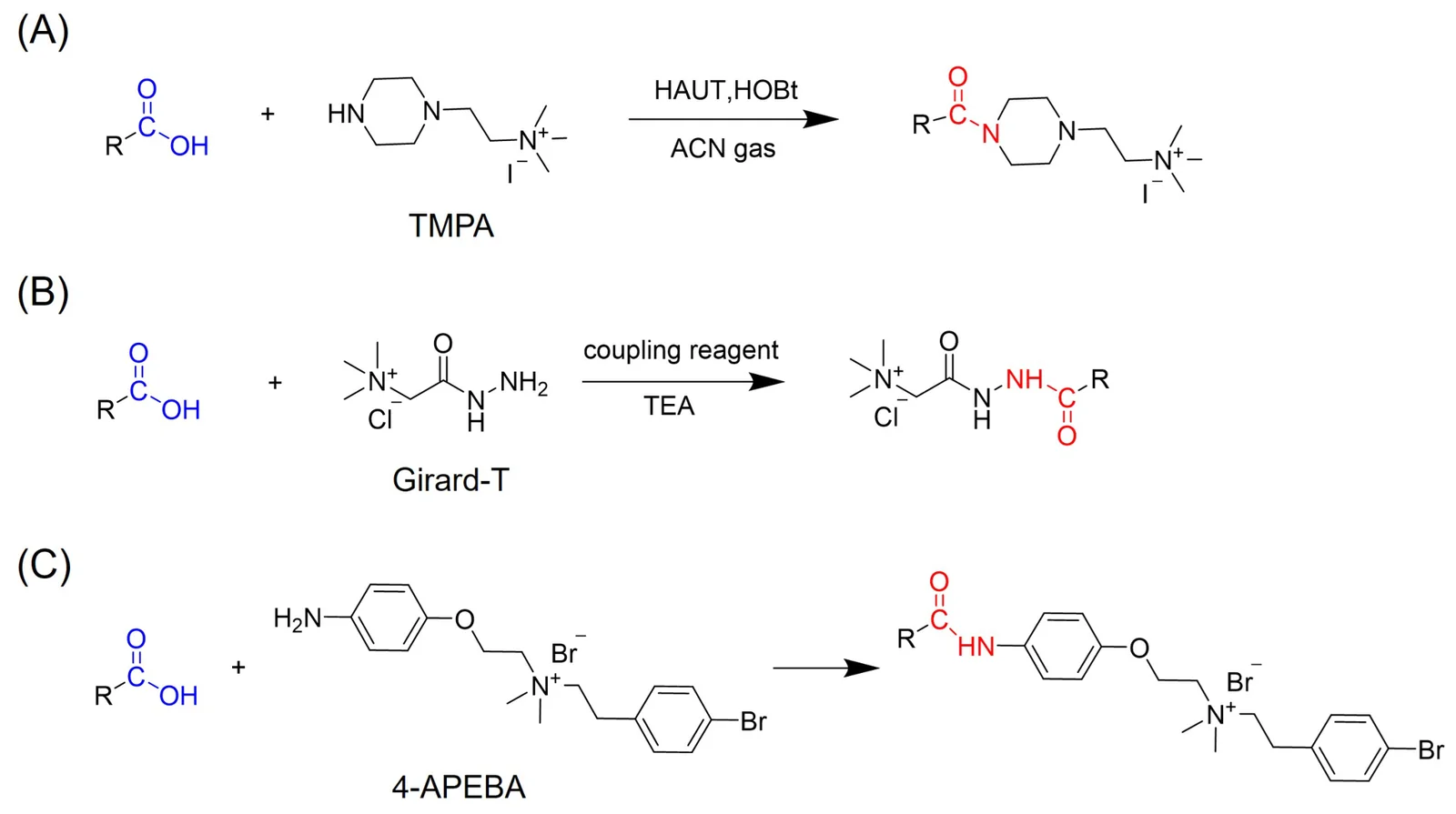
(**A**) N,N,N-trimethyl-2-(piperazin-1-yl)ethan-1-aminium iodide (TMPA)-mediated derivatization of carboxyl-containing metabolites. TMPA (3 mM), 1-[bis(dimethylamino)methylene]-1H-1,2,3-triazolo[4,5-b] pyridinium 3-oxide hexafluorophosphate (HATU; 1.5 mM), and 1-hydroxybenzotriazole (HOBt; 1.5 mM) in 90% ACN were spray-applied to the sample surface, followed by incubation in ACN vapor at room temperature for 4 h [16]. (**B**) Girard T-mediated derivatization of fatty acids. Girard T and 2-chloro-1-methylpyridinium iodide (CMPI) (10 mM each in 70% ACN) were spray-applied to the sample surface at a nozzle temperature of 35 °C, followed by incubation in 10% TEA/70% ACN vapor for 1 h [48]. (**C**) 4-(2-((4-bromophenethyl)dimethylammonium)ethoxy)benzenaminium dibromide (4-APEBA) derivatization of carboxylic acids. Carboxyl groups were first activated with aqueous 1-ethyl-3-(3-dimethylaminopropyl)carbodiimide (EDC; 16.66 μg/cm^2^), followed by application of aqueous 4-APEBA (5.56 μg/cm^2^) using a two-step spray protocol [49]. The target reactive functional groups in the analytes are highlighted in blue, and the corresponding derivatized moieties in the products are highlighted in red.

In 2020, Sun et al. proposed N,N,N-trimethyl-2-(piperazin-1-yl)ethan-1-aminium iodide (TMPA) as a derivatization reagent for the MALDI-MSI analysis of small carboxylic acid molecules in biological tissues (Figure 3A) [16]. Under activating conditions, TMPA forms a stable amide bond with the carboxyl group and introduces a quaternary ammonium structure, thereby achieving charge conversion of the carboxylic acid molecule. This strategy converts acidic carboxylic acid molecules into derivatives with a higher positive ion response, thereby expanding the MALDI analytical capabilities for carboxylic acid metabolites such as organic acids and fatty acids. Beyond signal enhancement, derivatization strategies have also been extended toward quantitative analysis. Differential isotope-coded labeling has been applied to MALDI-MS profiling of endogenous fatty acids, enabling relative quantification through paired isotopically labeled derivatives [47]. Recently, Han et al. developed an isotope-coded tissue surface derivatization strategy for the quantitative MALDI-MSI analysis of short-chain fatty acids [17]. By introducing isotope labels with mass differences, this method enables signal correction across different samples and reduces quantitative biases arising from sample preparation, tissue matrix effects and ionization differences. Consequently, carboxylic acid derivatization has gradually evolved from primarily enhancing detection responses toward enabling more reliable quantitative analysis, including spatial quantification by MALDI-MSI.

Apart from short-chain fatty acids, carboxylic acid derivatization strategies have also been progressively extended to the analysis of other types of acidic metabolites. For example, Girard T can be coupled with activated carboxylic acids to form positively charged acylhydrazide derivatives, thereby improving the MALDI-MS detection sensitivity of fatty acids in tissue sections (Figure 3B) [48]. Related on-tissue derivatization methods have also been applied to the spatial visualization of short-chain fatty acids, free fatty acids, and energy metabolism intermediates such as lactate and pyruvate [48,50,51]. In addition, 4-(2-((4-bromophenethyl)dimethylammonium)ethoxy)benzenaminium dibromide (4-APEBA) derivatization introduces both a permanent positive charge and a bromine-containing tag through amide bond formation with carboxyl groups, which has been used to expand the detectable coverage of polar plant metabolites in complex samples (Figure 3C) [49]. These studies demonstrate that carboxylic acid derivatization has expanded from the analysis of specific fatty acids to the study of multiple classes of acidic metabolites, providing new tools for analyzing the spatial distribution of metabolites in tissues.

For carboxylic acid metabolites, derivatization is particularly valuable because many acidic molecules show weak responses in positive-ion MALDI-MS. Fixed-charge labeling and isotope-coded strategies have improved the detectability and spatial quantification of carboxyl-containing metabolites, including short-chain fatty acids, free fatty acids and energy metabolism intermediates such as lactate and pyruvate. However, carboxyl activation and coupling reactions can be influenced by molecular structure, which may lead to differences in derivatization efficiency among carboxylic acids. Therefore, reaction reproducibility, isotope internal standards and standardized data processing are especially important for improving the quantitative reliability and spatial interpretability of MALDI-MS analysis in this class of metabolites.

### 3.5. Derivatization of Unsaturated Lipids and Lipid Isomers

Lipids are one of the most commonly analyzed classes of small molecules in MALDI-MS, yet they are structurally diverse and often contain numerous isomeric forms. At any given exact mass, there may be multiple isomers differing in fatty acid chain composition, the position of the C=C double bond, the number of double bonds, and cis-trans configuration. Consequently, the primary value of lipid double-bond derivatization lies not in general signal enhancement, but in converting otherwise inaccessible double-bond positional information into diagnostic fragments that can be resolved by MS/MS. This improves the ability of MALDI-MS to distinguish lipid isomers, rather than simply detecting lipid species [13,52,53].

**Figure 4:**
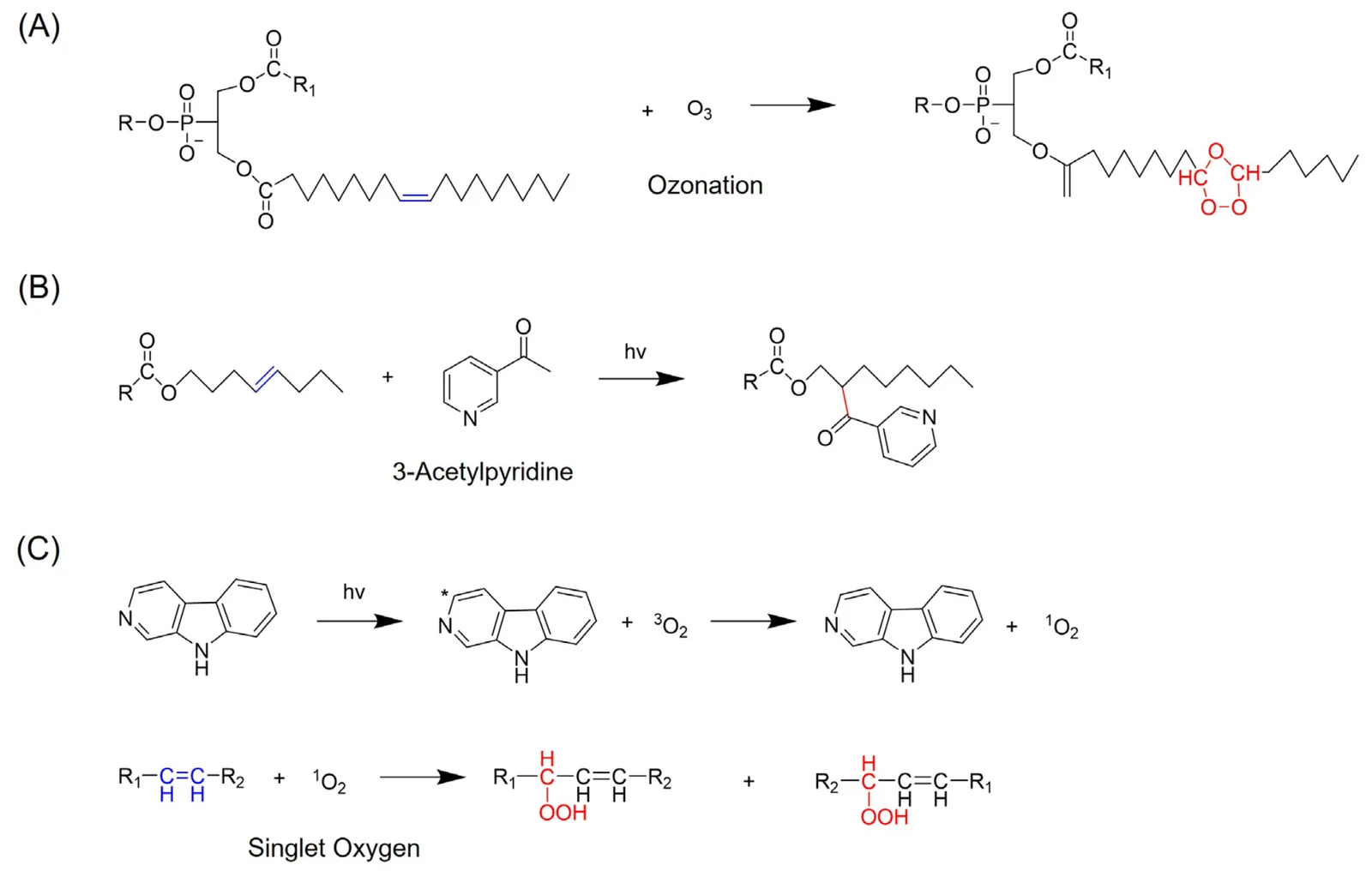
(**A**) Ozonation of unsaturated phospholipids. Dried samples were exposed to gaseous ozone in a glass chamber for up to 30 min [19]. (**B**) 3-Acetylpyridine-mediated Paternò–Büchi derivatization of lipid C=C bonds. 3-Acetylpyridine (110 mg/mL in MeOH containing 1% TFA) was pneumatically spray-applied to the sample surface, followed by UV irradiation for 30 min [54]. (**C**) Norharmane-mediated photooxidation of unsaturated lipids. Norharmane (10 mg/mL in 90% MeOH) was spray-applied to the sample surface with the nebulizing gas at 60 °C under white-light irradiation and served as both the MALDI matrix and photosensitizer [34]. The asterisk (*) denotes the photoexcited state of norharmane. The target reactive functional groups in the analytes are highlighted in blue, and the corresponding derivatized moieties in the products are highlighted in red.

Ozonation is an important derivatization strategy for localizing C=C double bonds in lipids. Bednařík et al. utilized ozonation of tissue sections to achieve MALDI-MS imaging of phospholipid C=C positional isomers (Figure 4A) [19]. Ozone reacts with the C=C double bonds in unsaturated lipids to generate ozonation products that indicate the position of the double bond, allowing the direct observation of the spatial distribution of isomers at different C=C positions within the tissue. By encoding lipid structural differences into characteristic fragmentation patterns, this strategy enables the discrimination of lipid isomers that cannot be resolved by MS1 alone and expands the application of MALDI-MS imaging in spatial lipid analysis.

The Paternò–Büchi (PB) reaction is another important method for the derivatization of lipid double bonds. This reaction utilizes a photo-induced [2+2] cycloaddition between a carbonyl compound and a lipid C=C double bond. Following MS/MS fragmentation, the products generate characteristic ions related to the position of the double bond. Chen et al. proposed a 3-acetylpyridine-mediated PB derivatization strategy on tissue surfaces for the resolution of lipid C=C positions via MALDI-MSI (Figure 4B) [54]. Unlike conventional lipid imaging, which merely reveals the spatial distribution of lipid molecules, this strategy further provides information on the localization of double bonds, enabling the distinction of different unsaturated lipid isomers within tissues at the structural level. The improvement in analytical coverage here mainly refers to expanding the range of lipid classes for which C=C bond localization and structural annotation can be achieved, rather than simply increasing the total number of detected lipids.

A variety of C=C reaction strategies have further expanded the scope of lipid isomer resolution. For example, cycloaddition and epoxidation reactions on tissue surfaces have been employed for the analysis of lipid double-bond positions, providing complementary structural information through different reaction products and fragmentation patterns [14,55]. These methods demonstrate that lipid double-bond derivatization has evolved from isolated derivatization reactions into an integrated analytical system in which multiple chemical reactions work together to resolve lipid structures. In recent years, another significant trend in lipid double-bond derivatization has been the integration of the reaction process with the MALDI-MS detection workflow. Rensner et al. proposed OzMALDI, incorporating the ozonation reaction into the MALDI ion source. In this system, double-bond reactions occur concurrently with mass spectrometric detection, which streamlines the workflow for lipid double-bond localization [33]. Additionally, the light-reactive norharmane strategy uses norharmane as both the MALDI matrix and the photoreactive reagent, allowing it to participate in the derivatization of lipid isomers under laser irradiation (Figure 4C) [34].

Alongside the development of chemical derivatization strategies, advances in mass spectrometry instrumentation and analytical methodologies have led to more reliable and precise discrimination of lipid isomers. For example, MALDI-2 technology promotes the secondary ionization process, thus improving the detection sensitivity of low-abundance derivatized lipids. Ion mobility mass spectrometry, meanwhile, adds an additional dimension of gas-phase separation, offering new possibilities for distinguishing lipid isomers with the same molecular weight but different structural features [56,57]. These advances have transformed lipid double-bond analysis into a comprehensive analytical system comprising structural labeling via chemical reactions, enhanced detection capabilities and structural identification.

Lipid double-bond derivatization differs from many conventional derivatization strategies because it primarily aims to improve structural discrimination rather than simply enhance signal intensity. Reactions such as ozonation and the PB reaction encode double-bond positional information into diagnostic fragments, enabling MALDI-MS to distinguish lipid isomers that cannot be reliably resolved by MS1 analysis alone. However, interpretation of derivative products remains challenging, particularly for polyunsaturated lipids with multiple reactive sites. Further advances will require improved reaction selectivity, high-resolution MS/MS, ion mobility separation, authentic standards, and curated lipid databases to achieve more reliable annotation of lipid double-bond isomers.

### 3.6. Derivatization Strategies for Additional Functional Groups and Molecular Classes

Derivatization strategies have also been developed for other functional groups, including thiols and phosphate groups, as well as for selected drugs and natural products, to improve their detection and molecular characterization by MALDI-MS. Given the significant structural diversity of these molecules, derivatization strategies are typically designed around specific chemical reaction sites, including the recognition of phosphate groups, and the structural annotation of complex molecular skeletons.

**Figure 5:**
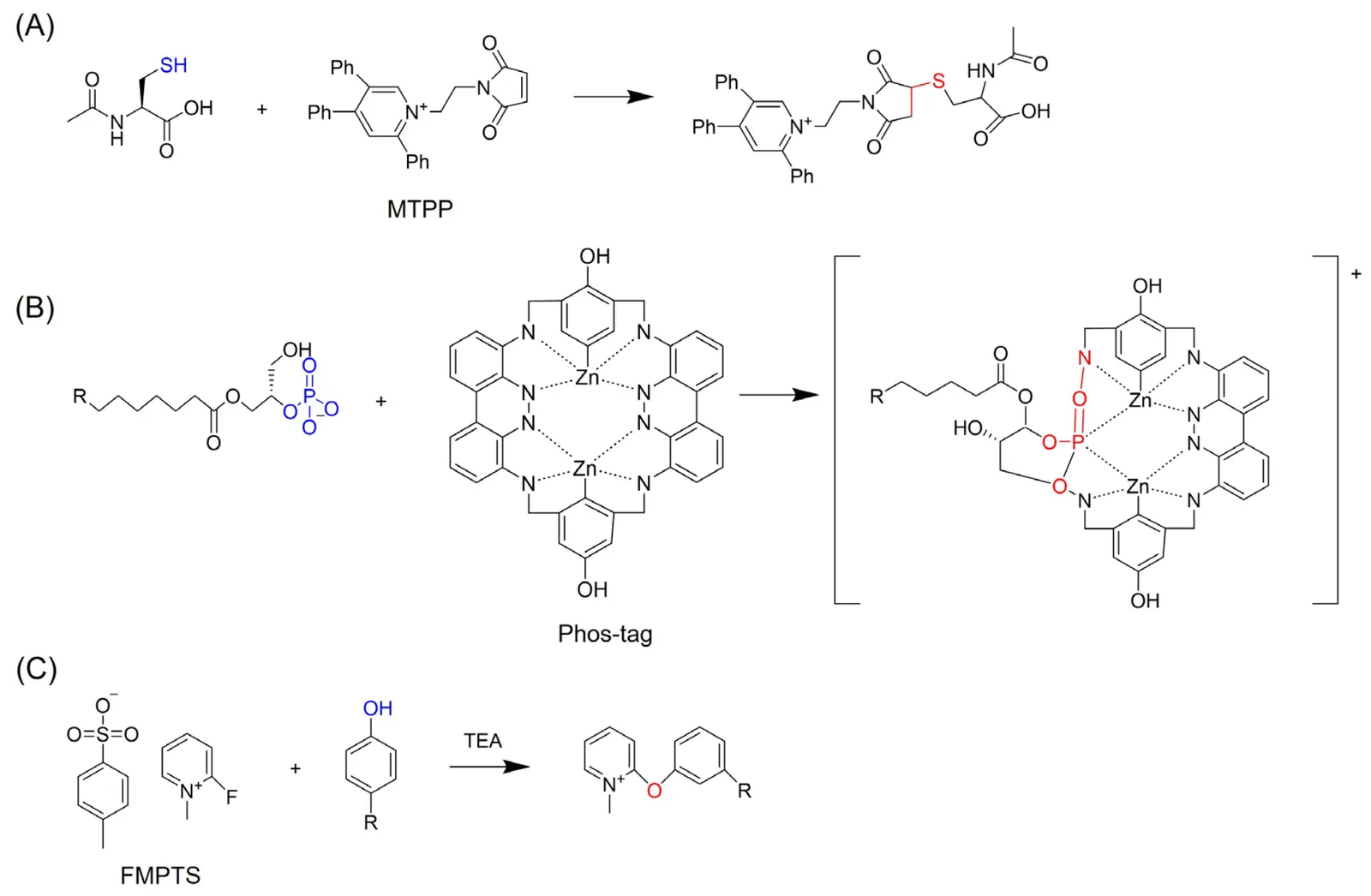
(**A**) 1-(2-(2,5-dioxo-2,5-dihydro-1H-pyrrol-1-yl)ethyl)-2,4,5-triphenylpyridinium (MTPP)-mediated derivatization of free thiols through thiol–maleimide addition. MTPP was applied to the sample surface and the labeling reaction proceeded at room temperature [59]. (**B**) Phos-tag-mediated recognition of phosphate monoester-containing lipids. An aqueous Phos-tag solution (0.86 mg/mL) was spray-applied to the sample surface before MALDI-MS analysis [60]. (**C**) 2-fluoro-1-methylpyridinium p-toluenesulfonate (FMPTS)-mediated derivatization of phenolic hydroxyl groups. FMPTS was applied to the sample surface in the presence of TEA as a base before MALDI-MS analysis [62]. The target reactive functional groups in the analytes are highlighted in blue, and the corresponding derivatized moieties in the products are highlighted in red.

Thiol-targeted derivatization has a distinct analytical role because it is closely related to the selective detection of redox-active molecules. Rather than merely enhancing the MALDI-MS response, this strategy can selectively capture molecules in defined redox states. Fülöp et al. developed the maleimide reagent (E)-2-cyano-N-(2-(2,5-dioxo-2,5-dihydro-1H-pyrrol-1-yl)ethyl)-3-(4-hydroxyphenyl)acrylamide (CHC-Mal) for the MALDI-MSI detection of free thiol metabolites and protein thiols through a thiol–maleimide addition reaction [58]. This strategy links thiol selectivity with mass spectrometric signal enhancement, providing a useful approach for analyzing redox-related small molecules and thiol-containing biomolecules. A related thiol-targeted strategy was reported by Huang et al., who introduced the pyridinium reagent 1-(2-(2,5-dioxo-2,5-dihydro-1H-pyrrol-1-yl)ethyl)-2,4,5-triphenylpyridinium (MTPP) for labeling free thiol-containing molecules. As shown in Figure 5A, MTPP reacts with thiol groups through a thiol–maleimide Michael addition reaction and introduces a permanent positive charge, thereby enhancing the MALDI-MS detection of thiol metabolites such as cysteine [59].

For phosphate monoester-containing bioactive lipids, Iwama et al. proposed an on-tissue Phos-tag-based derivatization strategy (Figure 5B). This strategy relies on the selective recognition of phosphate monoester groups by Phos-tag, thereby improving the MALDI-MSI detection of lipid phosphates such as lysophosphatidic acid (LPA) and sphingosine-1-phosphate (S1P) and enabling their spatial visualization in tissue sections [60].

In the analysis of natural products and pharmaceutical compounds, derivatization can support the structural annotation of complex molecules and improve the visualization of their tissue distributions. Dreisbach et al. combined on-tissue chemical derivatization with molecular networking to enhance the identification and visualization of steroidal glycosides in tissue sections [61]. In another study, Yan et al. applied on-tissue derivatization to the MALDI-MS analysis of anthraquinones, extending this strategy to the spatial mapping of exogenous natural products and pharmaceutical compounds (Figure 5C) [62].

As the range of molecular classes targeted by derivatization continues to expand, reliable identification of derivatization products and minimization of false-positive annotations have become critical issues. Strategies such as deuterated reactive matrices, functional group-based filtering and hydrogen–deuterium exchange can improve annotation reliability by providing diagnostic isotope shifts, functional group constraints or information on exchangeable hydrogens [29,63].

The examples discussed above show that functional-group-selective derivatization extends MALDI-MS analysis to functional groups and molecular classes that are difficult to characterize by conventional MALDI-MS alone. Thiol-containing compounds, phosphate monoester-containing lipids, natural products and drug-related molecules each require different recognition or reaction mechanisms, indicating that derivatization strategies are becoming increasingly tailored to specific chemical features. For these more diverse targets, clear reaction rules and standardized criteria for identifying derivatized products are particularly important. Fragment correlation analysis, isotope labeling and validation with standards can further strengthen the structural interpretation of complex small molecules. The representative derivatization strategies discussed throughout Section 3 are summarized and compared in Table 1 according to their target functional groups, representative reagents, derivatization modes, analytical applications, major advantages, and limitations.

## 4. Outlook and Conclusions

Chemical derivatization has substantially expanded the scope of MALDI-MS for small-biomolecule analysis. By introducing fixed charges, selective reaction tags, characteristic mass shifts, or diagnostic fragments, derivatization can improve the detection of weakly ionizing analytes, enhance functional-group-selective detection, and provide additional evidence for molecular discrimination. Different derivatization strategies provide complementary solutions depending on the analytical objective, with some approaches primarily enhancing ionization efficiency, whereas others improve molecular discrimination, quantitative reliability, or spatial compatibility. These strategies have expanded the range of small biomolecules accessible to MALDI-MS, including neurotransmitters, carbonyl-containing metabolites, carboxylic acids, and lipid isomers, while supporting spatially resolved analysis in complex biological samples.

Despite these advances, several challenges remain. First, derivatization efficiency can vary substantially among analytes because of differences in functional-group accessibility, molecular structure, steric hindrance, and the local chemical environment. Incomplete reactions, by-product formation and multiple derivatization sites may therefore affect signal reproducibility and quantitative reliability. These problems are particularly evident for heterogeneous carboxylic acids and polyunsaturated lipids, whose derivatized products may produce complex spectral patterns.

Second, reliable identification of derivatized products remains difficult. Expected mass shifts alone are often insufficient to distinguish authentic derivatives from background signals, unexpected reaction products, or structurally related candidates. The limited availability of derivative standards, reference spectra, and curated databases further constrains molecular annotation, especially in untargeted tissue analysis.

Third, on-tissue derivatization requires balancing reaction efficiency with spatial fidelity. Reagent deposition, solvent composition, humidity, and incubation conditions can influence both labeling efficiency and analyte diffusion. Differences in tissue composition may also contribute to spatial heterogeneity in reaction and ionization efficiencies, thereby affecting quantitative comparison across tissue regions.

For quantitative MALDI-MSI applications, reliable spatial quantification depends on several critical factors, including consistent derivatization efficiency, control of matrix effects during ionization, and the availability of suitable internal standards for signal normalization. Future efforts should focus on developing more selective derivatization chemistries and improving their integration with MALDI-MS workflows. Reaction performance should be evaluated systematically in terms of conversion efficiency, side-product formation, product stability, and matrix effects rather than signal enhancement alone. Stable-isotope labeling strategies and appropriate internal standards will be important for improving quantitative accuracy, whereas authentic standards, high-resolution MS/MS, and ion mobility separation will further strengthen the identification of derivatized products. Establishing standardized protocols for reagent deposition, reaction conditions, data processing, and reporting will also be essential for enhancing interlaboratory reproducibility. Beyond biofluid analysis and tissue imaging, single-cell analysis represents a promising direction for MALDI-MS derivatization. Advances in thiol-selective labeling on other mass spectrometric platforms suggest that cell-compatible probes and low-volume derivatization could improve the detection of reactive, low-abundance metabolites and reveal cellular heterogeneity [64].

In summary, chemical derivatization has become an important strategy for expanding MALDI-MS analysis of small biomolecules. Further advances in reagent design, reaction control, and analytical integration are expected to improve sensitivity, molecular discrimination, and spatial fidelity, while extending derivatization-assisted MALDI-MS from biofluid analysis and tissue imaging toward single-cell applications. These developments will broaden its utility for characterizing molecular heterogeneity in complex biological systems.

## Figures and Tables

**Table 1:** Summary and comparison of representative chemical derivatization strategies for small-biomolecule analysis by MALDI-MS.

Target Functional Group	Representative Reagent	Derivatization Mode	Application	Advantage	Limitation	Ref.
Primary amines	2,4-Diphenylpyrylium (DPP)	Reactive matrix-assisted derivatization	MALDI-MS/MSI: Monoamine neurotransmitter imaging	Introduces a permanent positive charge and enhances the MALDI response of primary amines	Restricted to compounds containing accessible primary amine groups	[15]
Primary amines	Bromopyrylium (Br-TPP)	Reactive matrix-assisted derivatization	MALDI-MSI: Monoamine neurotransmitter identification	Enhances ionization and provides a characteristic bromine isotope pattern for more confident identification	Primarily applicable to primary-amine-containing compounds	[35]
Amines and phenolic hydroxyl groups	4-(anthracen-9-yl)-2-fluoro-1-methylpyridin-1-ium iodide (FMP-10)	Reactive matrix-assisted derivatization	MALDI-MSI: Neuroactive compound mapping	Introduces a permanent positive charge and enables coverage of multiple classes of neuroactive compounds	Reacts with several nucleophilic functional groups rather than being specific to primary amines	[12]
Primary amines	2-nitrobenzaldehyde (NBA)	Photo-/in-source derivatization	MALDI-MS/MSI: Primary-amine metabolite analysis	Enables rapid nanosecond-scale photochemical labeling and close integration with MS analysis	Limited to analytes containing accessible primary amine groups and requires photochemical irradiation	[24,30,31]
Primary amines	5-methoxy-2-nitrobenzaldehyde (5-OCH_3_-NBA)	Photo-/in-source derivatization	MALDI-MS: Primary-amine neurometabolite profiling	Couples rapid covalent labeling with laser desorption/ionization and reduces low-mass background interference when combined with TiO_2_ P25	Requires coordinated use of the photo-reagent, photocatalytic matrix, and laser irradiation; primarily targets primary amines	[32]
Aldehydes and ketones	Girard’s reagent T (Girard T)	In situ derivatization on tissue surfaces	MALDI-MS/MSI: Corticosteroid imaging and quantification	Forms positively charged hydrazone derivatives and improves positive-ion detection	Derivatization efficiency may vary among different carbonyl compounds	[40]
Reducing-end carbonyl groups	Girard’s reagent P (Girard P)	In situ derivatization on tissue surfaces	MALDI-MS/MSI: N-glycan imaging	Introduces a positively charged pyridinium group and enhances N-glycan signals	Requires a released glycan with an accessible reducing-end carbonyl group	[41]
Aldehydes and ketones	Hydrazide-based reactive matrices	Reactive matrix-assisted derivatization	MALDI-MSI: Aldehyde and ketone imaging	Integrates carbonyl derivatization and MALDI matrix function within a single reagent system	Target scope is primarily restricted to aldehydes and ketones capable of reacting with hydrazide groups	[28]
Carbonyl groups	Parallel Girard T/Girard P derivatization	In situ derivatization on tissue surfaces	MALDI-MSI: Carbonyl metabolite annotation	Provides complementary reagent-specific mass shifts and spatial-distribution information for carbonyl assignment	Requires parallel derivatization experiments and comparison of reagent-specific signals and spatial distributions	[23]
Carboxyl groups	N,N,N-trimethyl-2-(piperazin-1-yl)ethan-1-aminium iodide (TMPA)	In situ derivatization on tissue surfaces	MALDI-MSI: Carboxyl-containing metabolite imaging	Introduces a quaternary ammonium group and improves positive-ion response of acidic metabolites	Requires activation and coupling of accessible carboxyl groups under controlled reaction conditions	[16]
Short-chain fatty acid carboxyl groups	Isotope-coded TMPA derivatization	In situ derivatization on tissue surfaces	MALDI-MSI: Short-chain fatty acids (SCFAs) quantification	Isotope coding provides internal correction and improves quantitative reliability in tissue analysis	Requires appropriate isotope-coded internal standards and consistent derivatization and reagent deposition	[17]
Carboxyl groups	Girard T	In situ derivatization on tissue surfaces	MALDI-MS/MSI: Fatty acid imaging	Formation of positively charged derivatives markedly improves fatty-acid detection sensitivity	Requires activation of carboxyl groups and coupling under basic reaction conditions	[48]
Carboxyl groups	4-(2-((4-bromophenethyl)dimethylammonium)ethoxy)benzenaminium dibromide (4-APEBA)	In situ derivatization on tissue surfaces	MALDI-MSI: Phytocompound imaging	Introduces a permanent positive charge and a bromine-containing isotope tag, facilitating sensitive detection and recognition	Carboxyl derivatization requires prior activation, and applicability depends on the presence of reactive target groups	[49]
Lipid C=C bonds	Ozone	In situ derivatization on tissue surfaces	MALDI-MSI: C=C positional-isomer imaging	Converts C=C positional information into characteristic ozonation products and diagnostic fragments	Requires ozone treatment and subsequent interpretation of reaction-derived product ions	[19]
Lipid C=C bonds	3-Acetylpyridine	Photo-/in-source derivatization	MALDI-MS/MSI: C=C positional-isomer imaging	Generates characteristic MS/MS fragments that enable localization of lipid double-bond positions	Requires photochemical derivatization followed by tandem MS for C=C positional assignment	[54]
Lipid C=C bonds	Methyltrioxorhenium (MTO)/ urea hydrogen peroxide (UHP)/ hexafluoroisopropanol(HFIP) system	On-tissue derivatization	MALDI-MSI: C=C positional-isomer imaging	Efficient epoxidation converts C=C bonds into products that provide diagnostic structural information upon fragmentation	Requires the combined epoxidation reagent/catalyst system and subsequent MS/MS analysis	[55]
Lipid C=C bonds	Ozone (OzMALDI)	Photo-/in-source derivatization	MALDI-MS/MSI: C=C position assignment	Integrates ozonolysis directly into the MALDI ion source without a separate derivatization-incubation step	Requires controlled introduction of ozone into the MALDI ion source	[33]
Lipid C=C bonds	Singlet-oxygen	Photo-/in-source derivatization	MALDI-MS/MSI: C=C positional-isomer imaging	Functions as both a MALDI matrix and photosensitizer, enabling singlet-oxygen-mediated lipid oxidation during analysis	Requires light-induced photosensitization and depends on the reactivity of unsaturated lipid C=C bonds	[34]
Thiol groups	(E)-2-cyano-N-(2-(2,5-dioxo-2,5-dihydro-1H-pyrrol-1-yl)ethyl)-3-(4-hydroxyphenyl)acrylamide (CHC-Mal)	In situ derivatization on tissue surfaces	MALDI-MSI: Free-thiol imaging	Selective labeling of free thiols with enhanced MALDI detection	Selective for accessible free thiols; oxidized sulfur forms are not directly labeled	[58]
Thiol groups	1-(2-(2,5-dioxo-2,5-dihydro-1H-pyrrol-1-yl)ethyl)-2,4,5-triphenylpyridinium (MTPP)	In situ derivatization on tissue surfaces	MALDI-MSI: Free-thiol metabolite imaging	Combines thiol-selective maleimide chemistry with permanent-charge labeling	Thiol oxidation before derivatization can reduce labeling efficiency	[59]
Phosphate monoesters	Phos-tag	In situ derivatization on tissue surfaces	MALDI-MS/MSI: Phosphate-monoester lipid imaging	Selective recognition of phosphate monoester groups enables detection of otherwise challenging phosphorylated lipids	Relatively narrow analyte coverage	[60]
Phenolic hydroxyl groups	2-fluoro-1-methylpyridinium p-toluenesulfonate (FMPTS)	In situ derivatization on tissue surfaces	MALDI-MSI: Anthraquinone imaging	Selective labeling of phenolic hydroxyl groups markedly improves MALDI-MS response of target compounds	Limited applicability to phenolic compounds	[62]

## Data Availability

No new data were created or analyzed in this study. Data sharing is not applicable to this article.

## Abbreviations

The following abbreviations are used in this manuscript:

LC-MS	Liquid chromatography–mass spectrometry
GC-MS	Gas chromatography–mass spectrometry
MALDI-MS	Matrix-assisted laser desorption/ionization mass spectrometry
MALDI-MSI	Matrix-assisted laser desorption/ionization mass spectrometry imaging
DPP	2,4-Diphenylpyrylium
Br-TPP	Bromopyrylium
FMP-10	4-(anthracen-9-yl)-2-fluoro-1-methylpyridin-1-ium iodide
NBA	2-Nitrobenzaldehyde
5-OCH_3_-NBA	5-Methoxy-2-Nitrobenzaldehyde
Girard T	Girard’s reagent T
Girard P	Girard’s reagent P
TMPA	N,N,N-trimethyl-2-(piperazin-1-yl)ethan-1-aminium iodide
4-APEBA	4-(2-((4-bromophenethyl)dimethylammonium)ethoxy)benzenaminium dibromide
PB	Paternò–Büchi
CHC-Mal	(E)-2-cyano-N-(2-(2,5-dioxo-2,5-dihydro-1H-pyrrol-1-yl)ethyl)-3-(4-hydroxyphenyl)acrylamide
MTPP	1-(2-(2,5-dioxo-2,5-dihydro-1H-pyrrol-1-yl)ethyl)-2,4,5-triphenylpyridinium
LPA	Lysophosphatidic acid
S1P	Sphingosine-1-phosphate
FMPTS	2-Fluoro-1-methylpyridinium p-toluenesulfonate
TFA	Trifluoroacetic acid
HATU	1-[bis(dimethylamino)methylene]-1H-1,2,3-triazolo[4,5-b]pyridinium 3-oxide hexafluorophosphate
HOBt	1-hydroxybenzotriazole
CMPI	2-chloro-1-methylpyridinium iodide
EDC	1-ethyl-3-(3-dimethylaminopropyl)carbodiimide
TEA	Triethylamine
MeOH	Methanol
ACN	Acetonitrile
